# Low dystrophin variability between muscles and stable expression over time in Becker muscular dystrophy using capillary Western immunoassay

**DOI:** 10.1038/s41598-021-84863-w

**Published:** 2021-03-15

**Authors:** Z. Koeks, A. A. Janson, C. Beekman, M. Signorelli, H. A. van Duyvenvoorde, J. C. van den Bergen, M. T. Hooijmans, I. Alleman, I. M. Hegeman, J. J. G. M. Verschuuren, J. C. v. Deutekom, P. Spitali, N. A. Datson, E. H. Niks

**Affiliations:** 1 Department of Neurology, Leiden University Medical Center, P.O. Box 9600, 2300 RC Leiden, The Netherlands; 2 BioMarin Nederland BV, Leiden, The Netherlands; 3 Department of Biomedical Data Sciences, Leiden University Medical Center, Leiden, The Netherlands; 4 Department of Human Genetics, Leiden University Medical Center, Leiden, The Netherlands; 5 Department of Clinical Genetics, Leiden University Medical Center, Leiden, The Netherlands; 6 Department of Radiology, Leiden University Medical Center, C.J. Gorter Center for High Field MRI, Leiden, The Netherlands; 7 Department of Physiotherapy, Leiden University Medical Center, Leiden, The Netherlands; 8 Duchenne Center Netherlands, Leiden, The Netherlands

**Keywords:** Biomarkers, Neurology

## Abstract

Becker muscular dystrophy (BMD) is the milder allelic variant of Duchenne muscular dystrophy, with higher dystrophin levels. To anticipate on results of interventions targeting dystrophin expression it is important to know the natural variation of dystrophin expression between different muscles and over time. Dystrophin was quantified using capillary Western immunoassay (Wes) in the anterior tibial (TA) muscle of 37 BMD patients. Variability was studied using two samples from the same TA biopsy site in nine patients, assessing nine longitudinal TA biopsies, and eight simultaneously obtained vastus lateralis (VL) muscle biopsies. Measurements were performed in duplicate with two primary antibodies. Baseline dystrophin levels were correlated to longitudinal muscle strength and functional outcomes. Results showed low technical variability and high precision for both antibodies. Dystrophin TA levels ranged from 4.8 to 97.7%, remained stable over a 3–5 year period, and did not correlate with changes in longitudinal muscle function. Dystrophin levels were comparable between TA and VL muscles. Intra-muscle biopsy variability was low (5.2% and 11.4% of the total variability of the two antibodies). These observations are relevant for the design of clinical trials targeting dystrophin production, and may urge the need for other biomarkers or surrogate endpoints.

## Introduction

The *DMD* gene is among the largest genes in the human genome. It covers 2.2 Mb and is located on the short arm of the X-chromosome^1^. Mutations in the *DMD* gene cause both Duchenne and Becker muscular dystrophies (DMD and BMD, respectively)^2^. DMD is the most severe form of the disease with symptoms occurring early in life, consisting of delayed motor development, reduced strength, loss of ambulatory capacity during the second decade, dilated cardiomyopathy and a reduced life expectancy^3,4^. BMD is the milder form with a large variability in disease course ranging from nearly asymptomatic with an almost normal life span to severe muscle weakness starting in early childhood and wheelchair dependence in the late teens and early 20 s^5–7^. DMD has been classically defined as the result of mutations abolishing synthesis of the gene product dystrophin and its absence in muscle biopsies. Indeed, lack of dystrophin is diagnostic in cases where the mutation is not found^8^. A large body of literature reports absence of dystrophin signal on Western blot, although immunofluorescent analysis may in some cases show some trace of dystrophin and/or rare revertant fibers, depending on the mutation^9^. Mutations causing BMD are mostly in-frame deletions resulting in the synthesis of shorter semi-functional dystrophin, which is typically identified by reduced levels in both immunofluorescent and Western blot analysis^10–13^. With the development of therapeutic approaches that aimed to restore dystrophin expression (e.g. through antisense mediated exon skipping, gene therapy or stop codon readthrough), dystrophin quantification has become central to show the pharmacodynamic effects of these drugs. The availability of novel technologies based on both immunofluorescence analysis^14,15^ and Western blot^16^ has enabled the refinement of the experimental procedures and provided new ways to quantitatively assess dystrophin levels. Application of the highly sensitive and quantitative capillary Western immunoassay (Wes) showed that DMD patients produce trace levels of dystrophin in muscle biopsies up to 7% of healthy controls, and that patients with a BMD diagnosis could have dystrophin levels from low (~ 10% of healthy controls) up to 100% of healthy controls^16^. These data support a differentiation between DMD and BMD based on dystrophin levels with a potential threshold effect at about 10% dystrophin, but also challenge the classical dichotomization between DMD and BMD and demonstrate a more fluid distribution with some overlap across the dystrophinopathy spectrum and healthy controls.

Analysis of dystrophin from muscle biopsies has been performed inconsistently across different muscle groups, without paired observations enabling comparison of dystrophin levels within and between muscles^17^. It is currently also unknown whether dystrophin levels are stable over time. Disease progression in BMD is typically slow and longitudinal studies are usually not long enough to observe a sufficient number of events (such as loss of ambulation) to support convincing associations between dystrophin levels and clinical progression. Also, several attempts have been made to identify potential associations between dystrophin levels and clinical performance in BMD patients^18,19^, but a clear and linear relationship has not been found.

We present data from an observational longitudinal study in BMD patients showing how dystrophin levels are stable across muscles and over time, but strongly vary between patients independent of mutation type and age, denoting a patient-specific capacity to synthesize dystrophin.

## Materials and methods

### Inclusion of participants and muscle biopsies

Participants consisted of ambulant and non-ambulant men aged 18 years or older, registered with a BMD diagnosis in the Dutch Dystrophinopathy Database (DDD)^20^ and children form the Leiden University Medical Center (LUMC) outpatient clinic. Adult patients had participated in two BMD natural history studies, one performed in 2011 (referred to as the 2011 study) and one starting in 2014 (referred to as the 2014 study).

Recruitment of participants for the 2011 study has been described previously^19^. Muscle strength was assessed via bilateral measurement of handgrip, flexion and extension of the knee, hip and elbow using quantitative muscle strength testing (QMT) with the quantitative muscle assessment (QMA) system (Aeverl Medical LLC; www.QMASystem.com: Gainesville, GA USA). Maximal voluntary isometric contraction (MVIC) was calculated as described by Hogrel et al.^21^. Functional tests were not part of this study protocol.

Participants in the 2014 study were also recruited via the DDD^22^. This study had a follow-up of 4 years and consisted of the following yearly functional tests: the six-minute walk test, North Star Ambulatory Assessment (NSAA) and timed tests (10 m walk/run, rise from the floor and four step ascend and descend test) in ambulant patients, and the Performance of Upper Limb version 1.2 in all participants. Next, muscle strength was assessed similar to the 2011 study. All assessments were done by two observers with help of a physiotherapist, trained previously as part of a clinical trial protocol. All participants in this natural history study were asked permission for muscle biopsies to be taken from the right TA and/or vastus lateralis muscle (VL) either at baseline or year 1, on the same day after all functional and muscle strength assessments had been performed. We chose to biopsy the clinically more severely affected right VL muscle for intramuscular comparison of dystrophin levels. An exclusion criterium for these biopsies was the use of anticoagulants.

The muscle biopsies from the pediatric BMD patients taken as part of the diagnostical work-up were included if material was available after completion of all diagnostic procedures (i.e. residual tissue). Functional assessments in these children had not been performed at the same time as the biopsies and were therefore not included in this study.

The 2011 and 2014 study protocol were both approved by the LUMC medical ethical committee. The clinical data and human tissue have been obtained, stored and handled in strict accordance with relevant guidelines and regulations. Written informed consent was obtained from all study participants. The residual muscle biopsy tissue of the pediatric BMD patients followed in the outpatient clinic at the LUMC is defined as tissue obtained for diagnostic purposes and that is residual after the diagnosis. The use of residual muscle tissue was in accordance with the codes of conduct as outlined by the Federation of Dutch Medical Scientific Societies. Oral consent from the children’s legal representatives was obtained by their treating physician, which is in accordance to regulations of the Medical Research Involving Human Subjects Act regarding research with residual human tissue.

### Muscle biopsies and dystrophin quantification

All muscle biopsies were obtained using a conchotome and performed according to standard protocol^23^. The skin was infiltrated with lidocaine 1%. A 1.5–2 cm skin incision was made and the fascia penetrated with a scalpel blade. A total of 3–4 samples were collected through the same incision. All samples were inspected for size and quality, transferred for transport into a plastic specimen bottle and snap-frozen in 2-methylbutane cooled in liquid nitrogen and stored at − 80 °C at the pathology department of the LUMC, all within 30 min.

#### Protein lysate preparation from muscle biopsies

Protein lysates from muscle samples were prepared by sectioning or cutting off small pieces of in total ~ 5–10 mg, further referred to as sections. These sections were placed in a MagNA Lyser vial and 100 μl Protein Lysis Buffer (15% SDS, 75 mM Tris–HCl pH 6.8, 1 Protease Inhibitor Cocktail tablet (Roche/Sigma 04693159001)/8 ml; 5% β-Mercaptoethanol) plus approximately 15 MagNA Lyser beads (#03358941001, Roche) were added. After briefly spinning down, the sections were then homogenized by 2–4 cycles (20 s; 7000 rpm) in the MagNA Lyser Instrument (Roche 03358976001) and spun down for 5 min at 13,000 rpm. The supernatant was supplemented with glycerol (final concentration 20%) and then lysates were stored at − 80 °C until further use.

**Figure 1:**
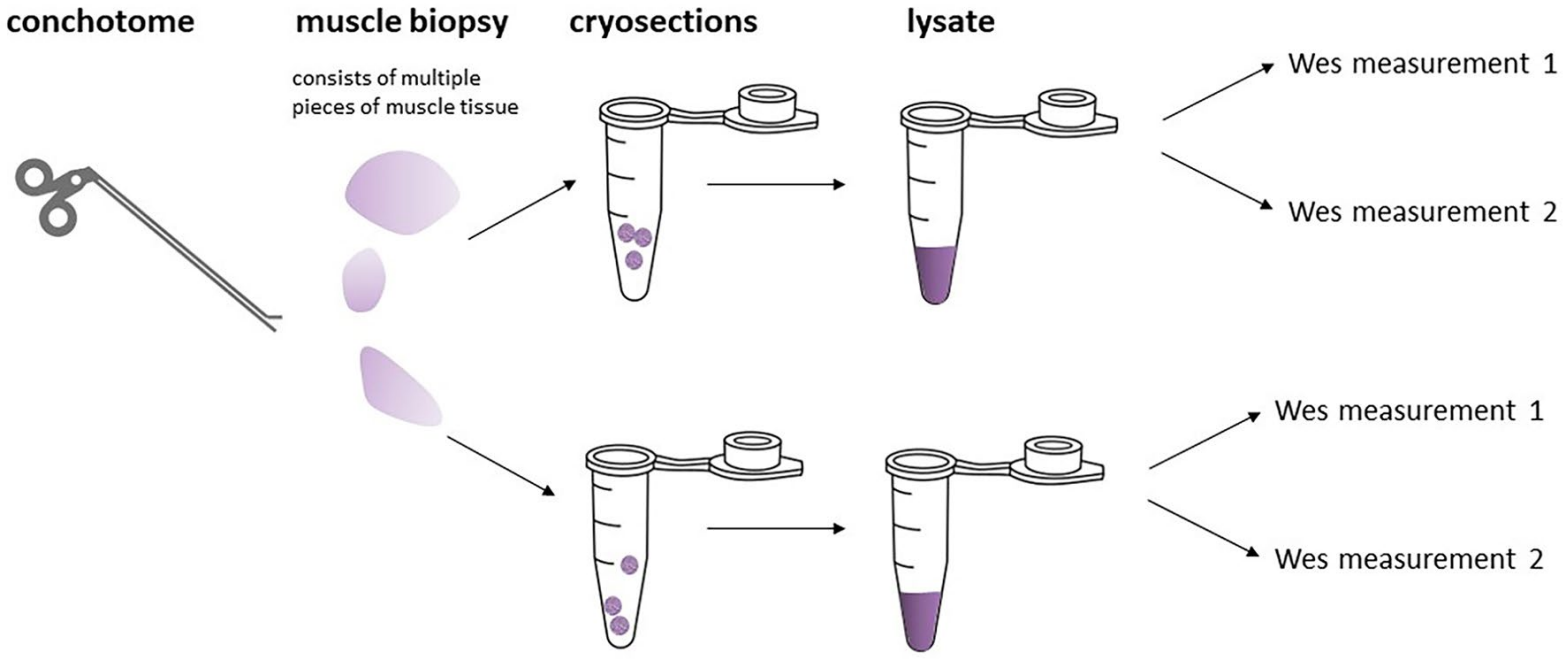
Illustration to explain the various steps and nomenclature from the biopsy procedure using a conchotome (performed in either the TA and/or the VL muscle), via 3–4 samples taken per biopsy and frozen on site, to sections cut in the laboratory, and finally the preparation of lysates that were all analyzed in duplo.

To measure total protein concentration, 20 × dilutions of the lysates in H_2_O were measured using the Pierce 660 nm Protein assay (#226607, Thermo Scientific) with added Ionic Detergent Compatible Reagent (Thermo Scientific 22663), according to the manufacturer’s instructions. Sequential steps from a muscle biopsy performed in each muscle to the lysates included in the final analyses are depicted in Fig. 1.

#### Wes procedure (Dystrophin detection)

Capillary Western immunoassay (Wes) analysis was performed as previously described^16^ on a Wes system (#004-600, ProteinSimple) according to the manufacturer’s instructions using a 66–440 kDa Separation Module (#SM-W006 or #SM-W008, ProteinSimple) combined with the No secondary Detection Module (#DM-003, ProteinSimple). For dystrophin detection mouse monoclonal Mandys106 (Glen Morris, dilution 1/50) and a rabbit monoclonal anti-dystrophin antibody (#ab154168, Abcam, dilution 1/1000) were used. In addition, an antibody targeting α-Actinin (#ab68167, Abcam, dilution 1/100) was used to control for muscle content. For the rabbit-derived primary antibodies (#ab154168 and #ab68167) an anti-rabbit secondary antibody (#042-206, Protein Simple) was used. For Mandys106 detection an anti-mouse secondary antibody (#042-205 Protein Simple) was used.

The lysates were diluted to 25 μg/ml, resulting in a loading amount of 0.125 μg per well/capillary. All lysates were analyzed by Wes twice, in separate experiments. To control for differences in chemiluminescence signal between experiments, a 6-point calibration curve of a healthy muscle reference sample was routinely included, usually ranging from 0.008 to 0.25 μg. This reference sample was selected from a panel of healthy controls based on it displaying average dystrophin levels with both ab154168 and Mandys106 as previously described^16^. Wes runs were considered valid if the calibration curve displayed an R^2^ > 0.99, based on 4–6 points in a relevant concentration range.

For α-actinin the calibration curve also ranged from 0.008 to 0.25 μg of the same healthy control. Since α-actinin abundance was too high to get reliable chemiluminescence values from the same lysate concentration required to measure dystrophin (substrate depletion), we reduced HRP activity by diluting the standard ProteinSimple secondary anti-rabbit-HRP antibody with 1/60 unconjugated anti-rabbit antibody (#ab6702, Abcam). The final muscle content-corrected dystrophin values were generated by first expressing both the dystrophin and α-actinin signals as percentage of control (% CTRL) using the following formula:

$$\% \,{\text{CTRL}} = \frac{{{\text{sample}}\,{\text{ protein}}\,{\text{ equal}}\,{\text{ to}}\, \, \times \upmu {\text{g}}/{\text{ml}}\,{\text{ CTRL}}\, \, \left( {{\text{from }}\,{\text{cal}}.{\text{curve}}} \right)}}{{ \times \,\upmu {\text{g}}/{\text{ml}}\,{\text{ sample}}\,{\text{ loaded}}}}$$

and then dividing the dystrophin % CTRL value by the α-actinin % CTRL value.

### Statistical analysis

All statistical analyses were performed using R (version 3.6.0)^24^. Comparison of the values of repeated measurements of the same antibody on each lysate was performed using scatter plots and computing Pearson’s correlation coefficient; the measurement error was estimated using a linear regression model with both measurements of the same antibody as response and lysate as covariates (measurement error = 1 − R^2^).

Paired measurements of Mandys106 and ab154168 were compared using scatter plots and computing Pearson’s correlation coefficient; the average difference between the two antibodies was computed and its significance tested using Wilcoxon’s paired samples test.

Comparison of dystrophin levels from adjacent samples from the same biopsy was achieved visually with a bar plot and the proportion of biological variability attributable to the different samples was estimated using a linear regression model with measurements from both samples as response and patient IDs as covariates (biological variability due to the pieces = 1 − R^2^).

Comparison of dystrophin levels in TA and VL was visualized with a bar plot. The mean difference between the two muscles was estimated and then tested using Wilcoxon’s paired samples test.

Changes of dystrophin levels over time were visualized by comparing the levels of dystrophin in the same patient over a time period ranging between 4 and 5 years. The average yearly change was computed and tested using Wilcoxon’s signed rank test.

The association between changes in patients’ performance and baseline dystrophin measures was estimated using a linear mixed model^25^ with changes in performance from baseline as response, baseline dystrophin values as covariate and a patient-specific random intercept included to account for the correlation between repeated measurements from the same individual. The significance of the associations was tested using a T test with Satterwhite’s approximation for the degrees of freedom, as implemented in the R package lmerTest^26^.

## Results

### Participants and available muscle biopsies

From 28 participants of the 2011 study, stored lysates and/or muscle tissue from the right TA muscle were available and included for re-analysis. Twenty-six participants had an in-frame mutation in the *DMD* gene and one participant had an out-of-frame mutation (deletion exon 3–7) with a mild disease course (age at wheelchair dependence 45 years). In one participant, no mutation had been found in the past using MLPA and high resolution melting curve analysis. Diagnosis had been made elsewhere by an experienced neuromuscular specialist, based on reduced dystrophin levels in a diagnostic muscle biopsy, raised serum creatine kinase levels, and calf hypertrophy.

Of the thirty-eight BMD patients who consented for participation in de 2014 study, two participants in whom a BMD diagnosis had been made elsewhere based on clinical characteristics and a diagnostic muscle biopsy were excluded after re-analysis of a newly obtained muscle biopsy and sequencing of the *DMD* gene. A gene panel was performed in both and revealed a homozygous mutation in exon 21 of the *CAPN3* gene for the first and a homozygous mutation in exon five of the *ANO5* gene for the second. For 35 of the remaining participants the diagnosis was confirmed by an in-frame mutation in the *DMD*-gene and one participant (who also participated in the 2011 study) had an out-of-frame mutation (deletion exon 3–7).

Of the 36 participants in the 2014 study, 13 consented for one muscle biopsy of the right TA muscle and of these 13 participants, 8 consented for an additional muscle biopsy of the right VL muscle. Nine of the 13 participants had also participated in the 2011 study.

Additionally, diagnostic muscle biopsies taken from the right TA muscle of 5 children (median age 6 years; range 6–12 years) with BMD were also included. Diagnoses in these children were based on an in-frame mutation in the *DMD* gene in 3 children, and an exon 3–7 deletion with a mild disease course in 2 children.

**Figure 2:**
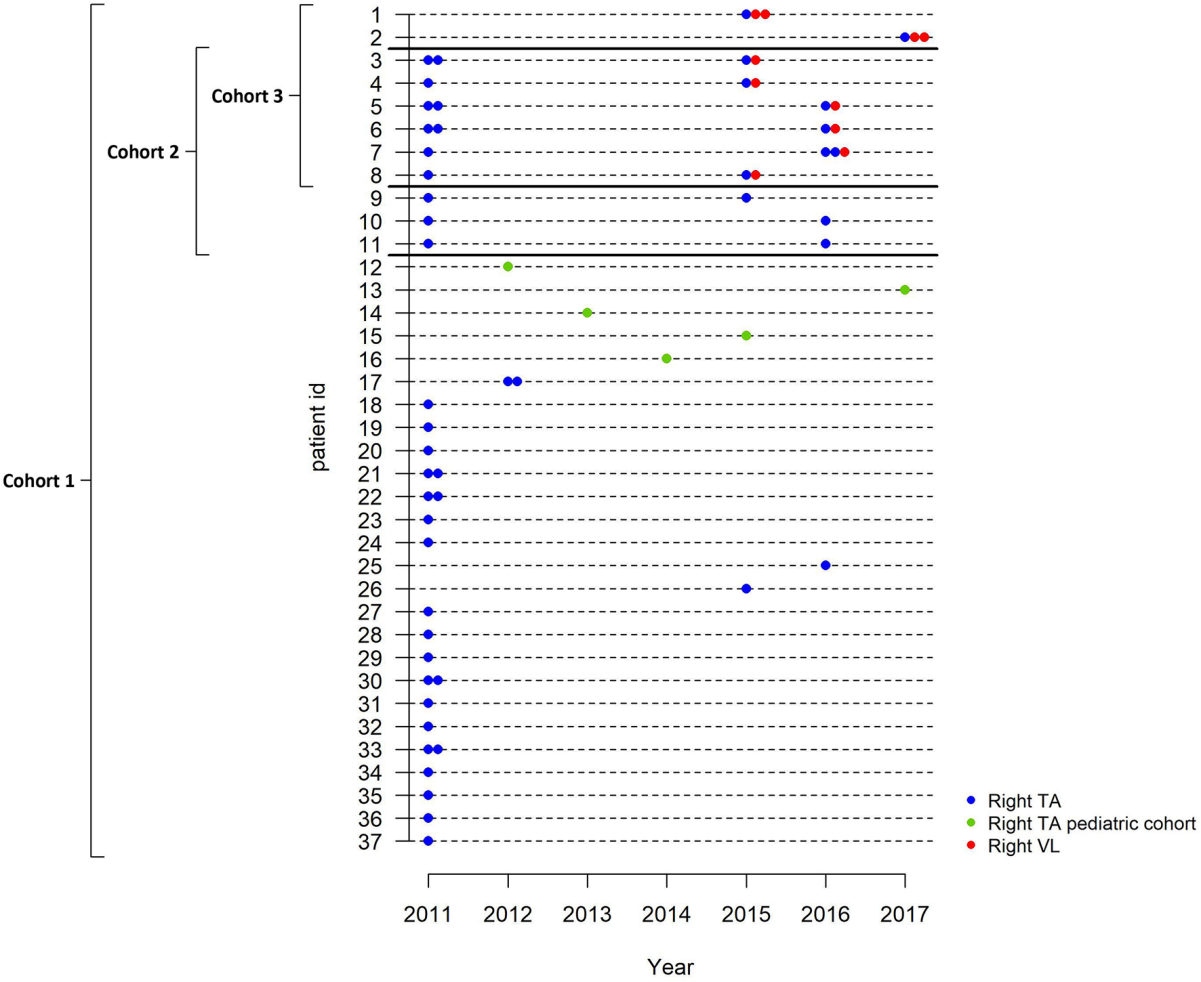
Overview of muscle biopsies and cohorts. Each horizontal line represents a study participant and participants are ordered by cohorts. Blue symbols represent biopsies obtained from the right tibialis anterior (TA) muscle of participants from either the 2011 or 2014 study, green symbols represent biopsies obtained from the right TA muscle from pediatric BMD patients, and red symbols represent biopsies obtained from the right vastus lateralis (VL) muscle of participants from the 2014 study. Two similar symbols indicate two samples analyzed from the same biopsy. Cohort 1 (cross-sectional analysis): participants of whom a biopsy from at least one point in time was available. Cohort 2 (longitudinal analysis): participants of whom a right TA biopsy was available from the 2011 and 2014 study. Cohort 3 (intermuscular analysis): participants from the 2014 study of whom biopsies formthe right TA and VL muscle obtained on the same day were available.

For the analysis participants were assigned into different study cohorts for analysis depending on their participation in one of the natural history studies or both, and type of muscle (TA and/or VL) biopsied (for an overview see Fig. 2).

*Cohort 1* (cross-sectional analysis) was defined by participants of whom a biopsy from at least one point in time was available: 28 from the 2011 study, 4 from the 2014 study, and 5 pediatric BMD patients adding to a total of 37 right TA biopsies for cross-sectional analysis.

*Cohort 2* (longitudinal analysis) consisted of 9 participants of whom a right TA biopsy was available from the 2011 and the 2014 study.

*Cohort 3* (inter-muscular analysis) was defined by 8 participants from the 2014 study of whom biopsies from the right TA and VL muscle were available obtained on the same day.

Finally, to allow comparison of dystrophin levels within the same muscle at the same point in time, two adjacent samples were assessed that had been taken from the same TA muscle site of 9 participants (8 participants from the 2011 and 1 of the 2014 study). Clinical characteristics of all participants per cohort are summarized in Table 1. Mutational data of all participants can be found in the Supplementary Table S1. None of the participants had ever used corticosteroids or were on (nocturnal) respiratory support.

**Table 1:** Patients characteristics for the three cohorts.

	Cohort 1 Cross-sectional analysis Right TA	Cohort 2 Longitudinal analysis Right TA	Cohort 3 Inter-muscular analysis Right TA vs Right VL
Number of participants	37	9	8
Age, years: median (range)	38 (1–66)	2011 study: 32 (21–48) 2014 study: 36 (25–52)	42 (25–59)
**DMD gene mutation type**^**a**^**: number (%)**			
Deletions	27 (73)	6 (66.7)	4 (50)
Duplications	3 (8.1)	1 (11.1)	2 (25)
Point mutations	6 (16.2)	2 (22.2)	2 (25)
No mutation found	1 (2.7)		
Ambulant^b^: n (%)	30 (81%)	9 (100%)	8 (100%)
Partial wheelchair use: n	3	0	1
Non-ambulant: n (%)	7 (19%)	0 (–)	0 (–)
Age loss of ambulation: median (range)	34 (11–57)	–	–
Cardiomyopathy: n (%)	13 (35%)	3 (33.3%)	2 (25%)

Cohort 1 (cross-sectional analysis): participants of whom at least 1 right TA muscle biopsy was available: 28 participants of the 2011 study, four participants of the 2014 study and five pediatric BMD patients from the outpatient clinic. Cohort 2 (longitudinal analysis): BMD participants of whom biopsy samples of the right TA muscle were available from the 2011 and 2014 study. Cohort 3 (inter-muscular analysis): participants from the 2014 study with biopsies of the right TA and VL muscle.

*TA *Tibialis anterior, *VL *Vastus lateralis.

^a^Detailed information on all mutations is shown in Supplementary Table S1.

^b^Ambulant was defined as being able to walk 10 m with support of a walking aid if needed.

### Dystrophin quantification

All dystrophin measurements were performed in duplicate with 2 independent antibodies, namely Mandys106 and ab154168 (see “Methods”). Dystrophin levels were below quantification in 1 TA and 3 VL muscles. Of the quantifiable samples, median level and range of absolute dystrophin levels in the TA using Mandys106 were 31.3% of control (CTRL) (4.8–97.7%) for cohort 1 and 33.0% of CTRL (21.6–97.7%) for cohort 2 at baseline (dystrophin % from biopsies of the 2011 study). In cohort 3, median dystrophin levels were 34.8% (18.6–86.4%) for the TA and 37.0% (12–103.4%) for the VL. In the five pediatric cases (part of cohort 1), dystrophin levels in the TA ranged from 4.8% to 18.1% and were lower than in the adults (range 12.5–97.7%) (Mann–Whitney U p < 0.001). Baseline dystrophin levels in the TA of all participants are displayed in Supplementary Table S1.

**Figure 3:**
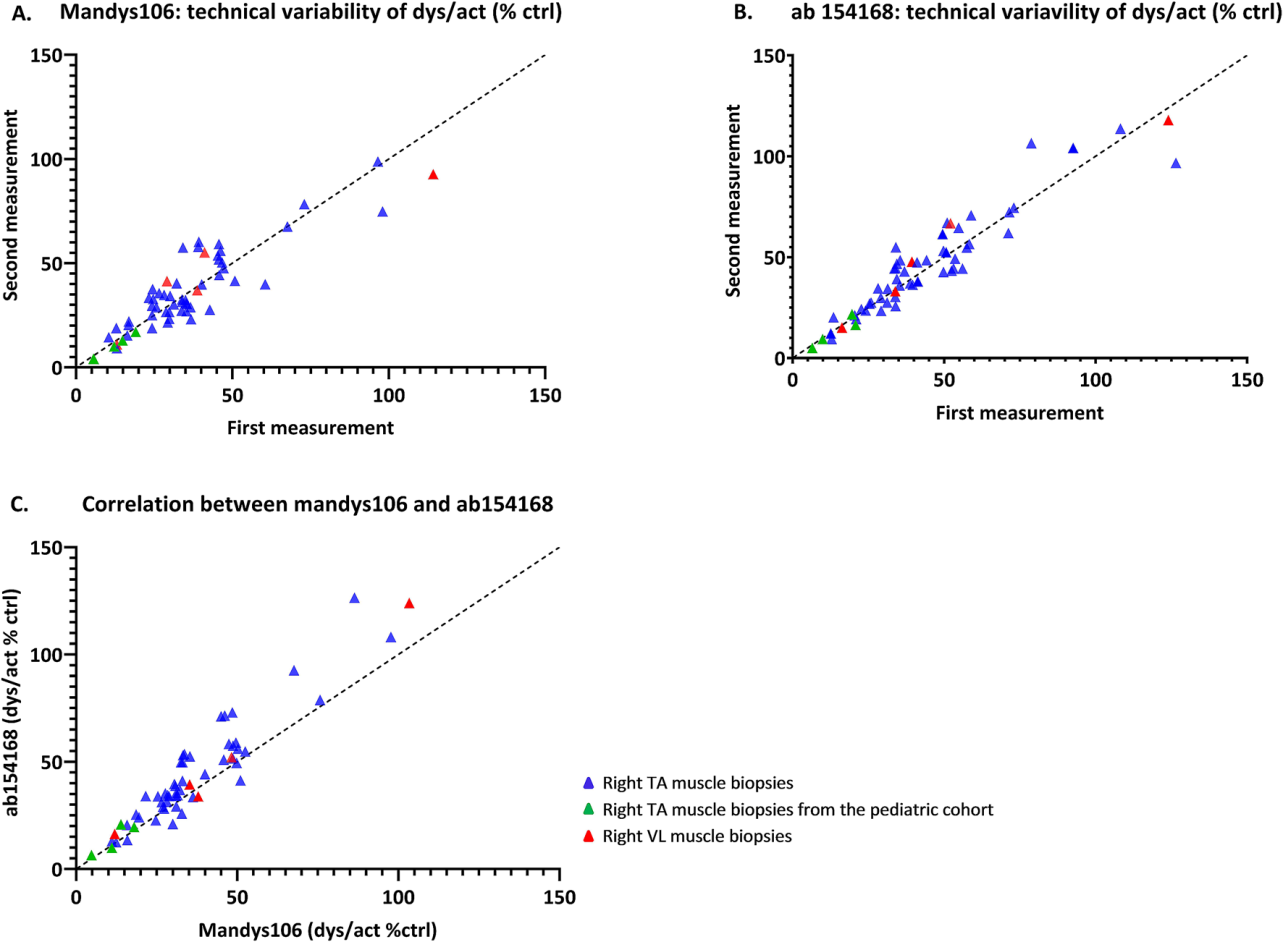
Dystrophin levels quantified with Mandys106 and ab154168. (**A**) Scatter plot showing the correlation between dystrophin quantification in two technical replicates with Mandys106 (Pearson’s correlation = 0.897; p < 0.0001). (**B**) Scatter plot showing the correlation between dystrophin quantification in two technical replicates with ab154168 (Pearson’s correlation = 0.940; p < 0.001). (**C**) Scatter plot showing the correlation between dystrophin quantification with Mandys106 and ab154168 (Pearson’s correlation = 0.972; p < 0.0001).

Dystrophin quantification showed a strong and highly significant correlation for measurements obtained with each antibody and across antibodies. The value of the Pearson’s correlation coefficient was equal to 0.897 (p < 0.0001, Fig. 3A) between replicates of Mandys106 and 0.940 (p < 0.0001) (Fig. 3B) between replicates of ab154168. Moreover, the correlation between the averaged values of Mandys106 and those of ab154168 was 0.972 (p < 0.0001) (Fig. 3C). The measurement error, estimated using the linear regression model, was found to be small for both antibodies. It accounted for 5.2% of the total variability of Mandys106 and for 3.1% of the total variability of ab154168. Dystrophin quantification by ab154168 yielded overall higher levels of dystrophin compared to quantification with Mandys106 (mean difference between ab154168 and Mandys106 = 7.9%, p-value of the paired samples Wilcoxon test < 0.0001).

**Figure 4:**
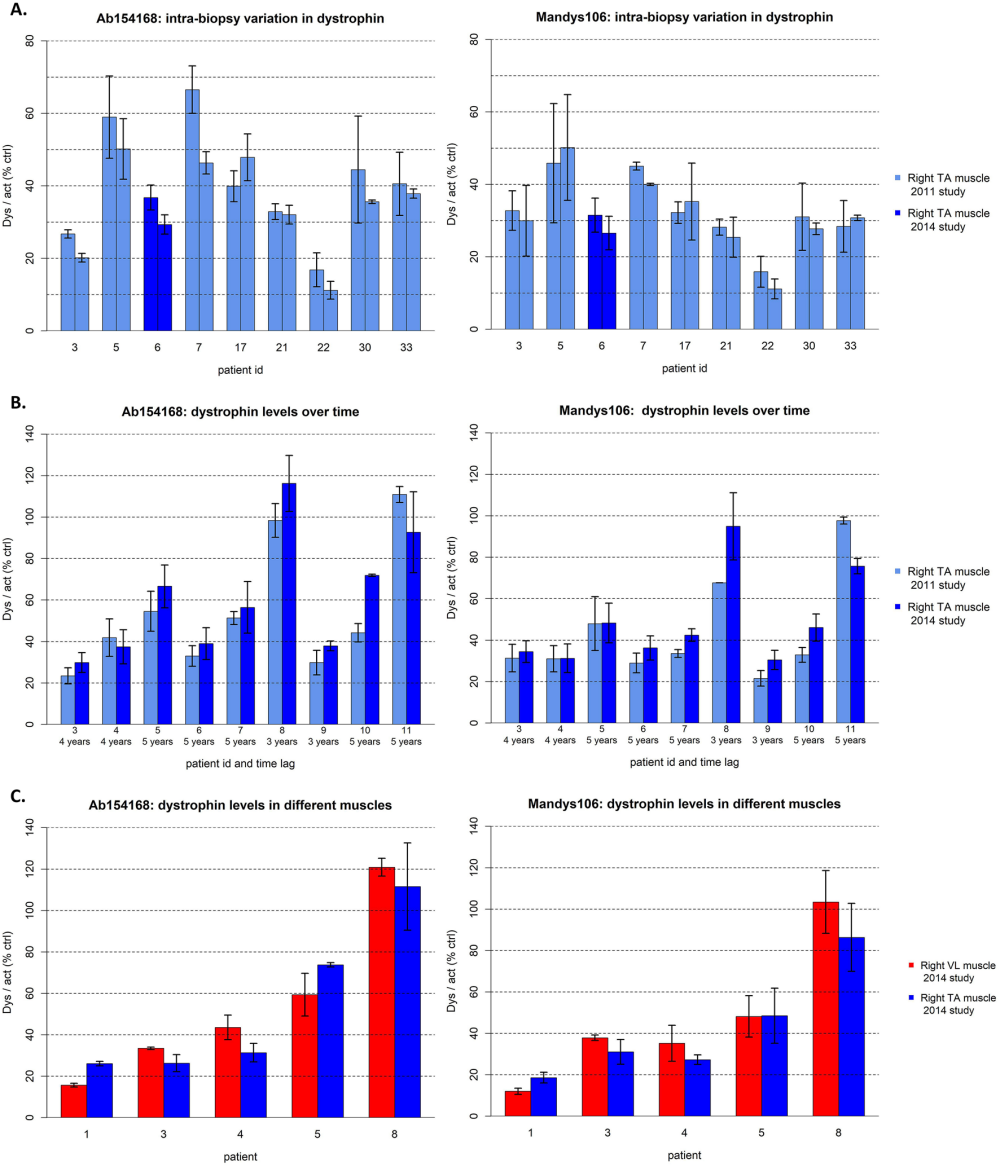
Variation in dystrophin levels. Bar graphs are presented as mean and standard deviation. (**A**) Bar graphs showing the mean and standard deviation of dystrophin levels quantified in two adjacent muscle samples obtained during the same biopsy procedure from the right TA muscle of participants from the 2011 (light blue bars) and 2014 (dark blue bars) study. (**B**) Bar graphs showing the mean and standard deviation of dystrophin levels over time [2011 (light blue bars) versus 2014 (dark blue bars) study] in right TA muscle samples. For each study participant the time lag between the two biopsy procedures are presented along the x-axis. (**C**) Bar graphs showing the mean and standard deviation of dystrophin levels of the right TA (blue) and right VL muscle (red).

Analysis of 2 adjacent muscle samples collected from the right TA at the same point in time (intra-biopsy analysis) showed a high correlation of dystrophin levels in all 9 study participants when analyzed with both antibodies independently (Fig. 4A). The biological variability was estimated to be equal to 5.2% of the total variability of Mandys106 and 11.4% of the total variability of ab154168.

In cohort 2, dystrophin was quantified in follow-up right TA biopsies from 9 participants obtained in the 2011 and 2014 study (biopsy procedure interval 3–5 years). Dystrophin levels in all biopsies were quantified twice with both antibodies for each given year. The mean and standard deviation of all measurements are displayed in Fig. 4B. Dystrophin levels were comparable across time for all participants. The average yearly change was not significant for both Mandys106 (average increase of 1.6%, p-value of the Wilcoxon signed rank test = 0.10) and ab154168 (average increase of 1.7%, p = 0.13).

In cohort 3, as expected the biopsy of the right VL was not informative for 3 out of 8 participants because it mainly contained fat, resulting in α-actinin levels below level of quantification. For the remaining 5 participants the analysis provided comparable levels of dystrophin for TA and VL biopsies. The average difference was not statistically different between TA and VL for both antibodies, Mandys106 (p-value of the paired samples Wilcoxon test = 0.84) and ab154168 (p = 0.84), respectively (Fig. 4C).

**Figure 5:**
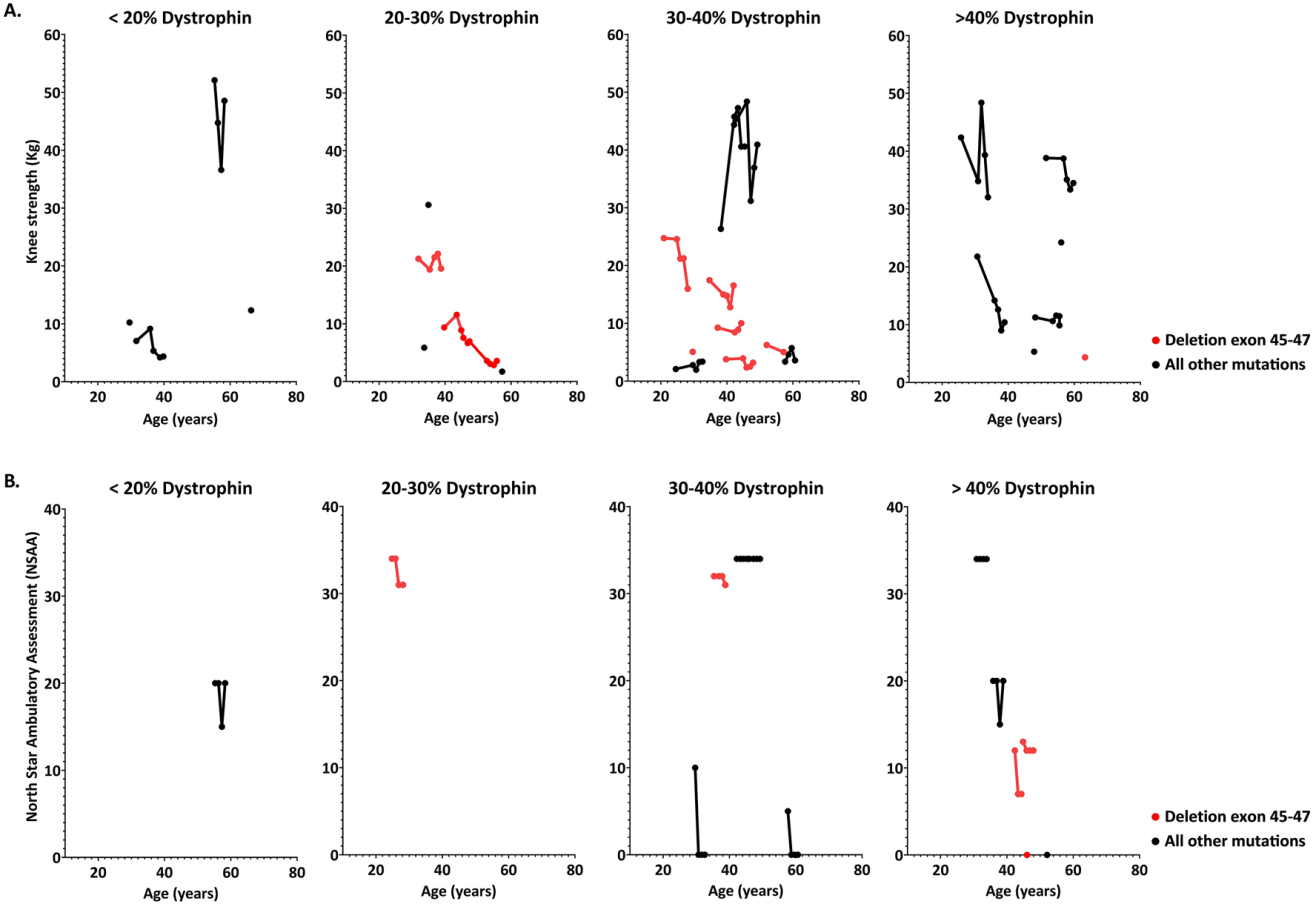
Association between baseline dystrophin levels and muscle strength and function. Scatter plots show individual assessments of change in knee strength and North Star Ambulatory Assessment (NSAA) over time. Longitudinal assessments within the same patients are represented by connecting the individual dots. The black dots represent patients with variable types of mutations and the red dots represent a subset of participants with a deletion of exon 45–47. Even in the deletion 45–47 group dystrophin levels and disease severity varied. (**A**) Change in knee strength over time grouped by dystrophin range. (**B**) Change in NSAA over time grouped by dystrophin range. NSAA results are limited as this test was only performed during the 2014 study.

To test whether yearly change in performance was affected by baseline dystrophin levels (either from the 2011 or the 2014 study, depending on when the first TA biopsy had been taken) we focused on knee extension as exemplar strength readout, and on the North Star Ambulatory Assessment (NSAA) as functional test. Available knee strength and NSAA outcome measures of participants organized by age and dystrophin range are presented in Fig. 5. Data of knee strength and NSAA were available for 24 and 13 participants respectively. Use of a linear mixed model allowed assessment of potential associations between TA dystrophin levels and performance changes. However, no significant association was found, irrespective of the antibody used to quantify dystrophin (knee extension: p = 0.66 using Mandys106 and p = 0.84 using ab154168; NSAA: p = 0.85 using Mandys106 and p = 0.66 using ab154168) (Fig. 5A,B).

## Discussion

In this study we assessed dystrophin levels in detail in a cohort of BMD patients with different mutations and variable disease severity. First, we showed that even though dystrophin levels vary considerably between BMD patients (ranging from 5 to more than 90%), their levels remain stable over a 3–5 year period. Second, dystrophin levels were comparable between two differently affected leg muscles, namely the right TA and VL muscle, as well as between independent samples obtained from the same TA muscle. Finally, we did not identify a clear relationship between dystrophin levels and changes in muscle strength (over 3–8 years of follow up) and function (over 3–5 years of follow up).

This is the first time that dystrophin levels have been analyzed longitudinally. Although results are from a limited number of samples, the fact that dystrophin levels remain stable over 3–5 years implies that progression of the disease is not caused by a progressive reduction in the dystrophin synthesis capacity.

The low variation in dystrophin levels observed between two differently affected leg muscles (TA and VL) collected during the same biopsy procedure suggests that, at least in the lower limbs, dystrophin levels do not determine the extent to which muscles are affected. However, due to the high fat infiltration observed in the VL, it is advisable to biopsy the more accessible and less affected TA muscle to obtain samples for protein quantification, even in severely affected patients. The availability of adjacent independent samples taken during the same biopsy procedure allowed to show that no differences in dystrophin levels are to be expected based on the sampling location. The overall lack of variation in dystrophin observed across muscles and over time raises new questions such as what factors are responsible for the individual-specific dystrophin levels and whether these apply to other proteins in muscle as well. Both *cis*- and *trans*-acting elements such as genetic variants, miRNAs and translation factors may be playing a role and deserve further research.

We also found that dystrophin levels remain relatively constant in stored muscle biopsies. We reanalyzed lysates prepared from stored (at − 80 °C) biopsies of a panel of 25 BMD patients from our previous study. Dystrophin levels were found to range from 10 to 90% of control in the previous study^16^ compared to 12–98% when reanalyzed for this study.

The lack of variation observed further supports the use of the Wes for dystrophin quantification. This technique used for dystrophin quantification has been proven to be highly sensitive, reproducible and quantitative over a large dynamic range with a lower limit of quantification; low enough to even quantify trace dystrophin levels^16^. Consistent with these findings, results in the present study confirmed this low technical variability. The percent coefficient of variation (CV%) between 2 replicate measurements of the same muscle lysate using 2 different anti-dystrophin antibodies was 5.2% for Mandys106 and 3.1% for ab154168, which is well below the accepted CV% for quantitative assays according to FDA/EMA regulations^27^. The advantage of the Wes high sensitivity is that it requires > 100-fold less muscle tissue compared to regular Western blot. Consequently, very small patient biopsies can be used including needle biopsies, instead of more invasive open biopsies that have a higher patient burden, especially in the pediatric population^28^.

At last, we also performed longitudinal muscle strength and functional tests to assess potential correlations between dystrophin levels, age and disease severity and progression. The disease severity in our BMD cohort showed the expected variability^5,6,19^. We were not able to demonstrate a direct relation between dystrophin levels and functional decline over a period of 3–5 years not even in the population with the same genotype (Deletion exon 45–47). This is in line with previous studies where no correlations between dystrophin levels and disease severity could be established when above 10–20% of normal^5,19^. Nonetheless, levels below 3–5% are still part of diagnostic criteria for DMD^11,16,19,29^ suggesting a dystrophin threshold that is relevant to the clinical phenotype. In the present study the lowest level of dystrophin among patients who were still ambulant by the age of 16 years was 12%. These dystrophin levels are still above those currently reported after longterm treatment with antisense oligonucleotides in DMD^30,31^. Despite the finding of relatively constant dystrophin levels over time, this level does not seem to be an adequate marker for disease severity or to predict the clinical course. Therefore there is a necessity to further explore disease modifying elements such as possible differences in regenerative capacity, epigenetic and/or environmental factors.

In summary, dystrophin expression can be quantified reliably by the state-of-the-art Wes capillary immunoassay and is relatively constant with an interval of 3–5 years in BMD patients with different disease severity. Sample variability within the same muscle and between the TA and VL muscle are both limited, although quantification is hampered in severely affected (VL) muscles due to fat infiltration. Similar to earlier studies, we were not able to demonstrate a relationship between dystrophin levels and age, nor between dystrophin and the rate of decline in muscle force or function, although there may be a threshold that is relevant to the dystrophinopathy phenotype. The large variability in dystrophin levels in BMD with a relatively slow progression in part of the patients, even over 3–5 years, is an important factor to consider in the development of clinical trials and may urge the need for other biomarkers or surrogate endpoints.

## Supplementary Information

Supplementary Table S1.
