# Supplementary material for: Low dystrophin variability between muscles and stable expression over time in Becker muscular dystrophy using capillary Western immunoassay
Source: Sci Rep. 2021 Mar 15;11:5952. doi: 10.1038/s41598-021-84863-w (PMC7971009; doi:10.1038/s41598-021-84863-w)
Supplement: Supplementary file 1 — Supplementary Table S1. [file 41598_2021_84863_MOESM1_ESM.pdf]

Low dystrophin variability between muscles and stable expression over time in Becker muscular dystrophy using capillary Western immunoassay

Z. Koeks[1], A.A. Janson[2], C. Beekman[2], M. Signorelli[3], H.A. van Duyvenvoorde[5,8], J.C. van den Bergen[1], M.T. Hooijmans[6], I. Alleman[7], I.M. Hegeman[1], J.J.G.M. Verschuuren[1,8], J.C. v. Deutekom[2], P. Spitali[4,8], N.A. Datson[2], E.H. Niks[1,8]

1. Leiden University Medical Center, Department of Neurology
2. BioMarin Nederland BV
3. Leiden University Medical Center, Department of Biomedical Data Sciences
4. Leiden University Medical Center, Department of Human Genetics
5. Leiden University Medical Center, Department of Clinical Genetics
6. Leiden University Medical Center, C.J. Gorter Center for High Field MRI, Department of Radiology
7. Leiden University Medical Center, Department of Physiotherapy
8. Duchenne Center Netherlands

Participant ID	Age at baseline	Study cohort	Mutation	TA dystrophin % baseline
1	55	1 + 3	Dup. 16-41	18.6
2	58	1 + 3	exon 6: c.419T>A p.(Leu140His) VUS	33.2
3	38	1 + 2 + 3	exon 29: c.3940C>T p.(Arg1314*)	32.8
4	21	1 + 2 + 3	Del 45-47	31.1
5	26	1 + 2 + 3	Del 48-49	45.8
6	25	1 + 2 + 3	Dup 02-07	31.5
7	37	1 + 2 + 3	Del 45-47	33.6
8	48	1 + 2 + 3	Del 45-48	67.6
9	32	1 + 2	Del 45-47	21.6
10	40	1 + 2	Del 45-47	33
11	31	1 + 2	exon 26: c.3515G>A p.(Trp1172*)	97.7
12	7	1	Del 3-7	4.8
13	6	1	Del 3-7	11.1
14	9	1	Del 45-46	13.9
15	12	1	Exon 4: c.244_246 dup p.(Arg82dup)	18
16	6	1	Intron 4: c265-2A>T	4.8
17	35	1	Del 45-47	32.2
18	34	1	Del 03-05	27
19	32	1	Del 03-04	19.5
20	47	1	Del 45-47	28.8
21	35	1	Del 45-47	28.2
22	20	1	Del 05	15.9
23	30	1	Del 45-47	33
24	52	1	Del 45-55	48.7
25	46	1	Del 45-47	52.5
26	46	1	Del 48-49	32.6
27	56	1	No mutation found	49.6
28	47	1	Del 45-47	47.4
29	66	1	Dup 14-42	15.8
30	52	1	Del 45-47	31
31	57	1	Del 3-7	24.7
32	50	1	Del 45-48	ND
33	40	1	Del 45-47	28.4
34	60	1	Del 45-48	49.8
35	30	1	DMD exon 19: c.2380+3A>C p.?	12.4
36	63	1	Del 45-47	51
37	48	1	Del 10-22	48.6
